# Unlocking the potential: exploring the impact of dolutegravir treatment on body mass index improvement in underweight adults with HIV in Malawi

**DOI:** 10.1186/s12889-024-18818-x

**Published:** 2024-05-16

**Authors:** Thulani Maphosa, Shalom Dunga, Lucky Makonokaya, Godfrey Woelk, Alice Maida, Alice Wang, Allan Ahimbisibwe, Rachel K. Chamanga, Suzgo B. Zimba, Dumbani Kayira, Rhoderick Machekano

**Affiliations:** 1 Elizabeth Glaser Pediatric AIDS Foundation, Lilongwe, Malawi; 2 Elizabeth Glaser Pediatric AIDS Foundation, Washington, DC USA; 3 Division of Global HIV and TB, U.S. Centers for Disease Control and Prevention, Lilongwe, Malawi

**Keywords:** Dolutegravir, Transition, Body mass index, Underweight, HIV, Malawi

## Abstract

**Background:**

The introduction of dolutegravir (DTG) in treating HIV has shown enhanced efficacy and tolerability. This study examined changes in weight gain and body mass index (BMI) at 6- and 12-months after post-initiating antiretroviral therapy (ART), comparing people living with HIV (PLHIV) on DTG-based regimens with those on non-DTG-based regimens in Malawi.

**Methods:**

Retrospective cohort data from 40 public health facilities in Malawi were used, including adult ART patients (aged ≥ 15 years) from January 2017 to March 2020. The primary outcomes were BMI changes/transitions, with secondary outcomes focused on estimating the proportion of mean weight gain > 10% post-ART initiation and BMI category transitions. Descriptive statistics and binomial regression were used to estimate the unadjusted and adjusted relative risks (RR) of weight gain of more than ( >) 10%.

**Results:**

The study included 3,520 adult ART patients with baseline weight after ART initiation, predominantly female (62.7%) and aged 25–49 (61.1%), with a median age of 33 years (interquartile range (IQR), 23–42 years). These findings highlight the influence of age, ART history, and current regimen on weight gain. After 12months follow up, compared to those aged 15–24 years, individuals aged 25–49 had an Adjusted RR (ARR) of 0.5 (95% Confidence Interval (CI): 0.35–0.70), suggesting a 50% reduced likelihood of > 10% weight gain after post-ART initiation. Similarly, those aged 50 + had an ARR of 0.33 (95% CI: 0.20–0.58), indicating a 67% decreased likelihood compared to the youngest age group 15–24 years. This study highlights the positive impact of DTG-based regimens, revealing significant transitions from underweight to normal BMI categories at 6- and 12-months post-initiation.

**Conclusion:**

This study provides insights into weight gain patterns in patients on DTG-based regimens compared with those on non-DTG regimens. Younger individuals (15–24 years) exhibited higher odds of weight gain, suggesting a need for increased surveillance in this age group. These findings contribute to the understanding DTG's potential effects on weight gain, aiding clinical decision making. Further research is required to comprehensively understand the underlying mechanisms and long-term implications of weight gain in patients receiving DTG-based regimens.

## Contributions to literature

- This study offers empirical evidence from a real-world setting, providing valuable insights into the effects of DTG-based regimens on weight and BMI changes in HIV patients.
- These practical data augment the theoretical knowledge derived from clinical trials, offering a more comprehensive understanding of the impact of DTG-based regimens.
- By highlighting the positive impacts on patients transitioning from underweight to normal BMI categories, this study addresses the potential treatment gap in HIV care.
- This study identified the potential risks associated with DTG-based regimens, such as the transition to higher BMI categories within a short duration.
- These findings emphasize the necessity for ongoing monitoring of patients on DTG regimens, particularly regarding weight gain and BMI changes.

## Introduction

Dolutegravir (DTG), a revolutionary HIV integrase strand transfer inhibitor (INSTI), is at the forefront of modern antiretroviral therapy (ART) owing to its unparalleled efficacy, exceptional tolerability, minimal drug interactions, and formidable resistance profile [1, 2]. Since the advent of generic once-daily fixed-dose combinations containing DTG (50 mg) alongside tenofovir and lamivudine (TLD) in September 2017, the landscape of HIV treatment has witnessed a transformative shift in clinical outcomes and affordability, with an astonishingly low annual cost of $75 per patient [1].

Clinical trials have unequivocally demonstrated the superiority of DTG-based regimens over traditional non-nucleoside reverse transcriptase inhibitor (NNRTI) regimens, showing higher rates of viral load suppression and reduced treatment discontinuation [1–3]. Notably, DTG surpasses the standard efavirenz (EFV) dose in terms of both efficacy and tolerability, boasting fewer drug interactions and a swifter onset of viral suppression [4–8].

In light of these remarkable advancements, numerous countries have orchestrated large-scale transitions to DTG-based ART for clinically stable individuals who have previously relied on EFV-based regimens. Malawi, a pioneer in HIV care, has been providing free ART since 2004 with approximately 828,000 people living with HIV actively receiving treatment. In January 2019, Malawi took a bold step forward by embracing DTG-based regimens as frontline therapies and replacing NNRTI-based regimens [9]. To date, almost all people living with HIV (PLHIV) in Malawi have transitioned to DTG-based regimens.

However, amidst the triumphs of DTG, concerns have arisen regarding its potential association with excess weight gain [8]. While weight gain can often signify a positive response to treatment, excessive gain raises concerns about the development of metabolic syndromes such as diabetes, cardiovascular diseases, and non-AIDS-related cancers [10]. Hence, our study aimed to investigate the effect of DTG-based regimens on weight and body mass index (BMI) at 6- and 12-month intervals after ART initiation. By contrasting the outcomes with those of non-DTG-based regimens during Malawi's transition program initiated in January 2019, we aspire to provide critical insights into the broader implications of DTG adoption in HIV care. Our investigation navigates the delicate balance between efficacy and potential adverse effects, shedding light on the multifaceted dimensions of modern ART strategies in resource-limited settings.

## Methods

We extracted retrospective cohort data from electronic and paper registers using routinely collected HIV treatment patient-level data from 40 health facilities in eight districts of Malawi. EGPAF-supported health facilities (*N* = 179) were stratified by district and patient volume (high: ≥ 1000 patients vs. low: < 1000 patients) to select 40 health facilities. The study facilities were randomly selected from each stratum to achieve a proportional representation. The study population included all patients (adolescents and adults aged ≥ 15 years) currently undergoing ART.

The data for this study were obtained from individual patient files/cards stored in health facilities. The data abstraction process occurred over two years before the implementation of the DTG rollout, specifically from 2017 to 2020. Stratified random sampling was used to select patients on antiretroviral therapy (ART) at EGPAF-supported sites between January 2017 and March 2020. Children below the age of 15 years were excluded from this study. Additionally, patient records with no recorded weights and those with data quality issues, such as extreme weight and height outliers, were excluded.

The primary objective of this study was to measure changes in body mass index (BMI) and other factors associated with > 10% weight gain at 6 and 12 months after ART initiation. The secondary objective was to compare the transition between BMI categories between patients receiving DTG-based regimens and those receiving non-DTG-based regimens.

### Statistical analysis

Descriptive statistics were used to summarize the demographic, clinical, and laboratory data. Categorical variables are presented as frequencies and percentages, and continuous variables are summarized using means and standard deviations or medians and ranges disaggregated by the DTG or non-DTG regimen. We estimated the proportion of ART-naïve and ART-experienced patients who initiated or transitioned to DTG-based regimens.

We estimated the proportion of patients experiencing > 10% weight gain from baseline to 6- and 12-months after post-ART initiation based on selected demographic and clinical characteristics, including sex, age group, ART history, and current treatment regimen. Binomial logistic regression was used to identify factors associated with > 10% weight gain. The 95% confidence intervals (CI) were calculated to quantify the precision of the estimated relative risks (RR), allowing for a comprehensive assessment of the association between the exposure variables and the outcome of interest while accounting for potential confounding factors. Body mass index (BMI) was categorized as underweight, normal weight, overweight, or obese. We further examined the changes in BMI categories at six months and 12 months from baseline in the DTG-based and non-DTG-based regimens. The frequency and proportion of patients in each baseline BMI category who transitioned to other categories were reported for each ART regimen group at six and 12 months.

### Ethical consideration

Permission and ethical clearance for this study were obtained from the Malawi National Health Science Research Council (NHSRC), Center for Disease Control and Prevention (CDC), and Advarra Institutional Review Board (IRB) in the United States. Since this was a retrospective study based on the abstraction of secondary data records, the ethics committees/institutional review boards that approved the protocol waived the need for informed consent.

## Results

**Fig. 1:**
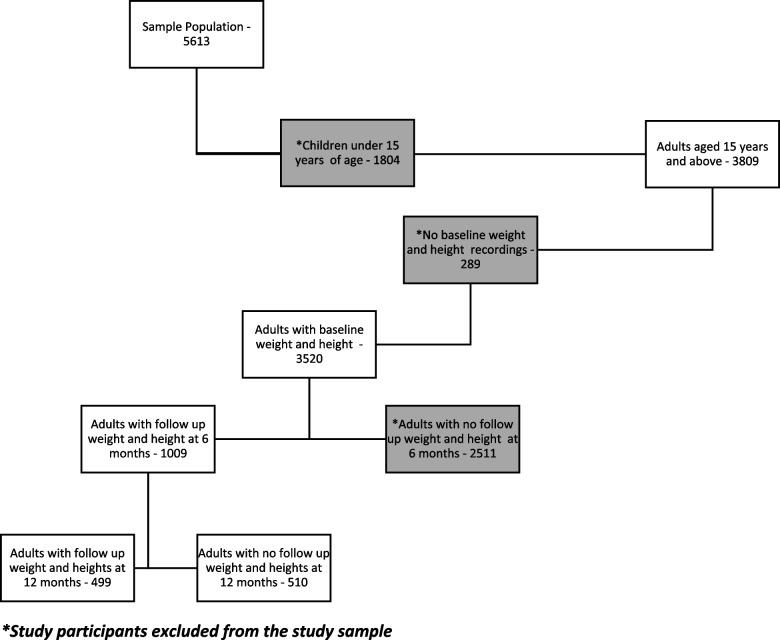
Flowchart showing patient eligibility for inclusion in the analysis

We reviewed the records of 5613 patients who were initiated or were already receiving ART between January 2017 and March 2020 in 40 selected public health facilities. Of the patients who initiated or were already on ART, 3809 (68%) were adults aged ≥ 15 years. Of the adults aged ≥ 15 years*, 3*520/3809 (92.4%) had baseline weight and height data within the study follow-up period, of whom 1009 and 499 had follow-up weight/height measurements at 6 and 12 months, respectively Fig. 1.

A total of 3209 (91.2%) patients were on a DTG-based regimen and 311 (8.8%) were on a non-DTG-based regimen during the study follow-up period. Among the study participants who received ART, 62.7% were female, with a median age of 33 years (interquartile range [IQR]: 24–42). Most patients (63.8%) were ART-naïve. The baseline BMI varied between the ART regimen groups, with 19.5% of the patients in the DTG group categorized as underweight compared to 43.1% in the non-DTG group Table 1.

**Table 1:** Characteristics of patients with a baseline weight and height on DTG and non-DTG-based regimens who were included in the analysis

**Baseline characteristics**			
	Patients on DTG n (%)	Patients not on DTG n (%)	Total N (%)
	***N* = 3209**	***N* = 311**	***N* = 3520**
**Gender**			
**Male**	1199 (37.4)	117 (37.6)	1316 (37.4)
**Female**	2010(62.6)	194 (62.4)	2204 (62.7)
**Age Group**			
**15-24yrs**	859(26.8)	128(41.3)	987(28.1)
**25-49yrs**	2002(62.4)	149(48.1)	2151(61.1)
**50 + yrs**	348(10.8)	33(10.7)	381(10.8)
**missing**		1	1
**ART History**			
**ART Experienced**	1156(36.0)	117(37.6)	1273(36.2)
**ART Naive**	2053(64.0)	194(62.4)	2247(63.8)
**Current ART Regimen**			
**NVP/EFV**	0	215 (69.1)	215(6.1)
**PI**	0	87 (27.7)	87(2.5)
**DTG**	3209(100)	0	3209(91.2)
**Other**	0	9 (2.9)	9(0.3)
**Baseline BMI**			
**Underweight**	604(19.5)	100 (43.1)	704(21.2)
**Normal**	2065(66.7)	117(50.4)	2182(65.6)
**Overweight**	340(11.0)	11(4.7)	351(10.6)
**Obese**	85(2.8)	4(1.7)	89(2.7)
**Excluded Weight or height or BMI outliers**	115	79	194
**Districts**			
**Blantyre**	749(23.3)	46(14.8)	795(22.6)
**Chiradzulu**	129(4.0)	23(7.4)	152(4.3)
**Dedza**	348(10.8)	27(8.7)	375(10.7)
**Mchinji**	358(11.2)	38(12.2)	396(11.3)
**Mwanza**	87(2.7)	4(1.3)	91(2.6)
**Ntcheu**	228(7.1)	28(9.0)	256(7.3)
**Thyolo**	971(30.3)	85(27.3)	1056(30.0)
**Zomba**	339(10.6)	60(19.3)	399(11.3)

A total of 180/1009 (17.8%) patients had a  > 10% increase in weight from baseline to 6 months post ART initiation. Six months after ART initiation, patients 25–49 years were less likely to experience > 10% weight gain (ARR = 0.56, 95% CI: 0.42 – 0.74) compared to those aged 15–24 years. In addition, treatment-naïve patients had a 32% higher likelihood of > 10% weight gain (ARR = 1.32, 95% CI: 0.99 – 1.76). After adjusting for age and treatment experience, the type of ART regimen was found to have no independent association with > 10% weight increase (ARR = 0.67, 95% CI: 0.52 – 1.12) Table 2.

**Table 2:** Factors associated with more than 10% weight gain at six months after initiating ART

Characteristic	N	> 10% weight gain N (%)	Unadjusted ARR (95%CI)	Adjusted ARR (95% CI)	*p*-value
Gender					
Male	396	68 (17.2%)	1	-	-
Female	613	112 (18.3%)	1.06 (0.81 – 1.40)		
Age group (years)					
15–24	324	80 (24.7%)	1	1	
25–49	531	72 (13.6%)	0.55 (0.41 – 0.73)	0.56 (0.42 – 0.74)	< 0.001
50 +	153	28 (18.3%)	0.74 (0.50 – 1.09)	0.80 (0.54 – 1.19)	0.275
**ART history**^a^					
Tx Experienced	719	117(16.3%)	1	1	
Tx Naive	290	63(21.7%)	1.32(1.01–1.76)	1.32 (0.99 -1.76)	
**Current regimen**^a^					
On Non-DTG regimen	99	25(25.3%)	1	1	
On DTG regimen	910	155(17.0%)	0.67(0.46–0.97)	0.67(0.52–1.12)	0.170

^a^Individuals who are treatment-naive and "On DTG regimen" have an ARR of approximately 2.567

-The *p* value for this interaction term was significant (*p* = 0.017)

At 12 months, of 155/499 (23.0%) patients exhibited a > 10% increase in weight from baseline, highlighting a notable trend in weight gain over time. Our findings show that the association between age and weight. Patients aged 25–49 (ARR = 0.50, 95%CI: 0.35 – 0.70) and those aged 50 + (ARR = 0.33, 95% CI: 0.20 – 0.58) were significantly less likely to experience > 10% weight gain compared to their younger counterparts aged 15–24. However, no statistically significant association was found among sex, ART history, current regimen, and > 10% weight gain Table 3.

**Table 3:** Factors associated with more than 10% weight gain at 12 months after initiating ART

Characteristic	N	> 10% weight gain N (%)	Unadjusted ARR (95%CI)	Adjusted ARR (95% CI)	*p*-value
Gender					
Male	241	53 (22.0%)	1	-	0.42
Female	258	62 (24.0%)	1.09 (0.79 – 1.51)		
Age group (years)					
15–24	141	55 (38.0%)	1	1	
25–49	243	45 (18.5%)	0.47 (0.34 – 0.66)	0.50 (0.35 – 0.70)	< 0.001
50 +	114	14 (12.3%)	0.31 (0.18 – 0.54)	0.33 (0.20 – 0.58)	< 0.001
**Missing**	1				
**ART history**					
Tx Experienced	443	96(21.7%)	1	1	
Tx Naive	56	19(33.9%)	1.56(1.04–2.35)	1.14 (0.70 -1.86)	0.60
**Current regimen**					
On Non-DTG regimen	56	22(39.3%)	1	1	
On DTG regimen	443	93(21.0%)	0.53(0.37–0.78)	0.68 (0.42–1.09)	0.09

The comparison of BMI category transitions between patients on DTG-based regimens (*N* = 910) and those on non-DTG-based regimens (*N* = 99) at six months revealed notable differences. Among the patients who were initially underweight, 59.2% (*n* = 135) in the DTG-based group remained underweight, compared to 81.4% (*n* = 35) in the non-DTG-based group. In the DTG-based regimen group, 85.0% (*n* = 476) of the individuals initially categorized as having a normal BMI maintained their status, in contrast to 79.1% (*n* = 34) in the non-DTG-based group at 6 months. At 12 months, among underweight individuals, 55.8% (*n* = 63) in the DTG-based group remained underweight compared with 90.0% (*n* = 17) in the non-DTG-based group. Within the normal BMI category, 81.4% (*n* = 223) of the DTG-based group and 63.6% (*n* = 21) of the non-DTG-based group remained unchanged. Among overweight individuals, 32.1% (*n* = 27) in the DTG-based group transitioned to normal BMI category, whereas 33.3% (*n* = 1) on the non-DTG-based group at 6 months. In obese individuals, 70.4% (*n* = 19) in the DTG-based group and 100% (*n* = 1) in the non-DTG-based group remained unchanged at 6 months Table 4.

**Table 4:** Comparison of the transition between BMI categories among patients on DTG-based regimens and patients on non-DTG-based regimens

	**BMI change at six months**							
	**DTG-based regimen group *N* = 910**				**Non-DTG-based regimen group *N* = 99**			
	**Underweight n (%)**	**Normal n (%)**	**Overweight n (%)**	**Obese n (%)**	**Underweight n (%)**	**Normal n (%)**	**Overweight n (%)**	**Obese n (%)**
**Underweight**	135(59.2)	92(40.4)	1(0.4)	0	35(81.4)	8 (18.6)	0	0
**Normal**	43 (7.7)	476(85.0)	40(7.1)	1(0.2)	8(18.6)	34(79.1)	1(2.3)	0
**Overweight**	1(1.2)	27(32.1)	46(54.8)	10(11.9)	0	1(33.3)	1(33.3)	1(33.3)
**Obese**	3(11.1)	0	5(18.5)	19(70.4)	0	0	0	1 (100)
	**BMI change at 12 months**							
	**DTG-based regimen group *N* = 443**				**Non-DTG-based regimen group *N* = 56**			
**Underweight**	63(55.8)	48(42.5)	1(0.9)	1(0.9)	17 (90.0)	4(19.1)	0	0
**Normal**	25(9.1)	223(81.4)	26(9.5)	0	8(23.5)	21(63.6)	4(12.1)	0
**Overweight**	0	11(29.0)	22(57.8)	1(13.2)	0	0	1(50)	1(50)
**Obese**	2(16.7)	1(8.3)	1(8.3)	8(66.7)	0	0	0	2(100)

In summary, at the monthly follow-up, approximately 40.4% of patients on DTG-based regimens who were initially underweight achieved a healthy weight. In contrast, only 18.6% of the patients on non-DTG-based regimens showed a similar transition (*p* = 0.006). Furthermore, at 12 months post-initiation, 42.5% of the patients on DTG-based regimens who were initially underweight attained a normal BMI, whereas this was the case for only 19.1% of those on non-DTG regimens (*p* = 0.051). Among patients with a healthy weight at DTG-based regimen initiation, 41 (7.3%) and 26 (9.5%) transitioned to an unhealthy weight at 6 and 12 months, respectively. Among patients with healthy weight at the initiation of a non-DTG-based regimen, one (2.3%) and four (12.1%) patients gained unhealthy weight.

## Discussion

Our analysis revealed compelling evidence for the beneficial effects of DTG-based regimens in patients. Specifically, a significant proportion of patients experienced transitions from underweight to normal BMI at both 6- and 12-month follow-up intervals, indicating improvements in nutritional status and overall health outcomes associated with DTG-based treatment regimens. Moreover, our analysis revealed noteworthy adverse effects in initially normal-weight patients, who progressed to overweight and obesity. These findings are consistent with existing research demonstrating a gradual increase in BMI among treatment-experienced cohorts [10–13]. In regions such as Malawi and other comparable settings, these insights are pivotal for enhancing HIV patient outcomes during the transition to and initiation of DTG therapy. Our investigation of the weight dynamics following antiretroviral therapy (ART) initiation uncovered notable patterns. Among the 1009 patients examined, 17.8% experienced remarkable weight gain exceeding 10% at 6-month milestone. As the journey progressed to the 12-month mark, our scrutiny intensified, revealing a persistent pattern: 23.0% of 499 patients showed substantial weight gain. These findings illuminate the evolving landscape of weight dynamics post-ART initiation, underscoring the significance of understanding and managing weight changes in HIV patients undergoing treatment. Furthermore, our findings suggest that factors such as age and treatment history may influence weight gain in patients receiving ART [10]. The lower likelihood of excess weight gain in the 25–49 age group and 50 + years age groups could be attributed to various factors, including differences in metabolic rates, lifestyle factors, and comorbidities [12, 14, 15]. Additionally, the higher proportion of excess weight gain among treatment-naive patients indicates that prior exposure to antiretroviral treatment may influence weight changes [16–18].

Our analysis did not reveal a significant correlation between the current regimen, particularly the DTG regimen, and excess weight gain, suggesting that DTG-based regimens may not exert a substantial influence on weight gain compared with non-DTG regimens [19]. Interestingly, the study observed a higher proportion of patients experiencing ≥ 10% weight increase while on DTG regimens at both 6 and 12 months, implying that weight gain is anticipated as patients receive more treatment irrespective of the regimen [19]. Our analysis further demonstrated a positive impact of DTG-based regimens on the health and well-being of patients, as evidenced by the significant proportion of patients transitioning from underweight to normal BMI at the 6- and 12-month follow-up. These findings align with those of studies showing gradual increases in BMI over time in treatment-experienced cohorts [9]. The change from underweight to normal or maintaining a higher proportion with normal BMI observed in patients on DTG-based regimens suggests an improved or maintanance of good nutritional status and overall health. Adequate nutrition is essential for individuals living with HIV as it plays a crucial role in supporting immune function, medication adherence, and overall quality of life. The positive impact of DTG-based regimens on BMI suggests that this treatment approach may contribute to better treatment outcomes and long-term health benefits in HIV patients [8, 13, 14].

Additionally, we examined the transitions between BMI categories. We observed that patients receiving DTG-based regimens were more likely to transition from normal to overweight and observed a significant proportion from transitioning from overweight to obese within the 6-month follow-up period. These results highlight the importance of ongoing monitoring of patients receiving DTG regimens, as there may be implications for increased mortality due to non-communicable diseases among individuals receiving DTG-based treatment [10, 12, 20]. The transition to higher BMI categories among patients receiving DTG-based regimens emphasizes the need for comprehensive healthcare interventions that address not only HIV treatment but also management of associated comorbidities [12].

While our study provides valuable clinical insights into the real-world impact of DTG-based regimens, it has several limitations that require careful consideration. Significant drop-offs in participant numbers at the 6- and 12-month follow-up intervals suggested a potential bias in relation to the main outcomes, highlighting the need for further investigation. Moreover, the absence of data on crucial factors such as rural/urban location, food security, socioeconomic indicators, and opportunistic infections limits the depth of our analysis and may have confounded our results. Additionally, the lack of information on transitions to DTG during the observation period adds complexity to our findings. Furthermore, suboptimal availability and quality of data on weight and height pose challenges, resulting in a notable loss of individuals available for BMI-related analyses during the 12-month follow-up period. Despite these constraints, our study underscores the urgency of addressing these limitations to enhance our understanding of the real-world implications of DTG use.

## Conclusions

In conclusion, our findings tentatively suggest the promising benefits of DTG-based regimens for weight gain and BMI improvement among patients undergoing ART [19]. However, caution is warranted due to the study's limitations, notably data attrition and absence of key covariates. Vigilant monitoring of patients on DTG regimens, particularly regarding weight gain and BMI changes, is imperative for the early detection of potential risks and implementation of targeted interventions to safeguard long-term health outcomes. Moving forward, robust research efforts are essential to confirm and elucidate these findings in larger and more diverse populations. Additionally, investigating the multifaceted factors influencing weight gain during DTG-based treatment will further enrich our understanding and enhance the clinical management strategies.

## Data Availability

The datasets used and/or analyzed during the current study are available from the corresponding author upon reasonable request. Access to data is subject to institutional and ethical restrictions.

## Abbreviations

ART: Antiretroviral therapy
BMI: Body Mass Index
CDC: Centers for Disease Control and Prevention
DTG: Dolutegravir
EGPAF: Elizabeth Glaser Pediatric AIDS Foundation
